# Occupational adaptation of retired elite athletes transitioning into school coaches in China

**DOI:** 10.1371/journal.pone.0358744

**Published:** 2026-10-05

**Authors:** Tianchen Zheng, Zhidong Dai

**Affiliations:** 1 School of Physical Education, Shanghai University of Sport, Shanghai, China; 2 School of Physical Education, Chuzhou University, Anhui, China; Instituto Politecnico de Viana do Castelo, PORTUGAL

## Abstract

**Background:**

The transition of retired elite athletes into high-performance or professional coaching has received considerable scholarly attention, whereas their transition into school coaching remains underexplored. In 2023, China established the position of school coach under the policy agenda of integrating sports and education, marking the emergence of a new occupation within the education system. This study explores the occupational adaptation process of retired elite athletes transitioning into school coaching positions, with the aim of promoting the career development and well-being of school coaches and providing new insights into the emergence and practice of new occupational roles within contexts of institutional transformation.

**Methods:**

This qualitative study, grounded in constructivist epistemology, conducted semi-structured interviews with 30 school coaches with elite athletic backgrounds from multiple regions in China (Anhui, Zhejiang, and Guangdong provinces). Purposive and maximum variation sampling were employed to capture diversity in participants’ demographic characteristics and professional backgrounds, including age, gender, athletic level, sport type, school level, geographic region. Interview data collected between March and December 2025 were analyzed using reflexive thematic analysis, with researcher reflexivity and continuous analytical discussions employed to enhance trustworthiness.

**Results:**

The occupational adaptation of retired athletes transitioning into school coaching positions unfolds through three stages: role pre-adaptation, role negotiation, and role orientation. During pre-adaptation, individuals formed preliminary understandings of the role through training and internships, though their perceptions remained influenced by competitive sport logic. After entering schools, they experienced reality shock and ongoing role negotiation as tensions emerged between competitive sport experiences and educational contexts. Over time, adaptation outcomes diverged into three forms: role integration, role assimilation, and role alienation.

**Conclusions:**

The occupational adaptation of school coaches is a dynamic process of occupational identity construction shaped by state logic, school logic, and professional logic, while also influenced by individual agency. The findings provide new insights into the literature on elite athletes’ career transition and occupational adaptation. Future efforts should further improve the working mechanisms and evaluation systems for school coaches in order to promote the institutionalization and professionalization of this occupation.

## Introduction

For a long time, the retirement and career transition of elite athletes has been regarded as a critical issue in the field of sport. Traditional perspectives have often treated athletes’ retirement as a relatively controllable or even natural termination of a career. However, recent empirical studies and social realities have increasingly shown that this stage is often accompanied by complex and profound challenges of adaptation [1]. Existing research suggests that retirement is not a singular event, but rather a transitional process characterized by uncertainty and tension, during which individuals must cope with multiple pressures within a short period, including identity transformation, the reconstruction of social relationships, and adjustments in lifestyle [2,3]. Consequently, scholarly attention has gradually shifted from a predominantly psychological perspective toward a more comprehensive socio-cultural approach, emphasizing how individuals continuously construct and reconstruct their identities through interaction within specific contexts [4]. Within this shift, career transition is understood as a dynamic process of adaptation between the individual and the environment. Building on Schlossberg’s transition theory, Stambulova proposed the Athletic Career Transition Model, which suggests that when facing transition demands, athletes must mobilize personal resources while coping with external barriers, leading to outcomes such as successful transition, delayed transition, or crisis transition [5]. Further studies have shown that the quality of transition is influenced by multiple factors, including athletic identity, autonomy in retirement decision-making, educational and economic conditions, and social support [6]. Meanwhile, the Holistic Athletic Career Model proposed by Wylleman and Lavallee reveals the complexity of athletes’ career development and transitions across multiple dimensions—athletic, psychological, social, academic, and vocational—emphasizing that transition is not merely a career shift but a systemic adjustment across the individual life course [7]. In recent years, research has further incorporated an ecological perspective, highlighting that athletes’ career transitions occur within the interaction of multiple environments. The concept of the “Transitional Environment,” proposed by Henriksen, Stambulova, and colleagues, conceptualizes the transition process as a dynamic system connecting the original athletic environment and the target occupational environment, within which individuals continuously adjust their cognition, behavior, and identity [8]. This perspective indicates that career transition is not only determined by individual capabilities but is also profoundly shaped by institutional structures, organizational culture, and social relationship networks. Supportive organizational cultures, role models, knowledge sharing, and empowerment mechanisms are considered key conditions facilitating successful transitions [9].

Among various transition pathways, the shift from athlete to professional coach has gradually attracted scholarly attention. Compared to entering non-sport occupations, transitioning into coaching involves a certain degree of “contextual continuity,” as athletic experience and skills can be partially transferred into the new profession, making adaptation relatively easier [10]. However, this transition process may still be highly challenging. Athletes are required to shift from a “self-performance-oriented” role to an “other-development-oriented” role, with success criteria moving from quantifiable results to more abstract educational and developmental outcomes [1,11]. Individuals must also reconstruct their professional identity, transforming from being “coached” to becoming “the coach,” a process involving complex meaning-making and identity reconstruction [12]. Although prior athletic experience constitutes an important resource, it is insufficient for fulfilling coaching responsibilities. Many novice coaches demonstrate clear deficiencies in pedagogy, communication skills, and knowledge of athlete development. From the perspective of development pathways, The transition from athlete to coach can be categorized into two types: “proactive” and “passive.” The former accumulate coaching experience and engage in career planning during their athletic careers, while the latter enter coaching positions passively after retirement, often without systematic preparation [13]. This distinction directly affects the smoothness of their adaptation process.

Another line of research has focused on the “fast-track” phenomenon in athlete-to-coach transitions, whereby athletes enter coaching roles rapidly based on their competitive achievements but without systematic training [14,15]. Although athletic experience is often regarded as a valuable asset, empirical studies show that athletes entering coaching roles through fast-track pathways often lack experience in pedagogy, communication, and team management, and thus face multiple challenges in the early stages of their careers, including redefining career motivation, acquiring new knowledge systems, and adjusting interaction patterns with others [16,17]. Some studies further point out that the “elite mindset” developed during athletes’ careers may become a barrier in coaching contexts—for example, difficulties in understanding athletes of different skill levels or in translating embodied experience into teachable knowledge [15]. The transition from “I do” to “I teach” thus represents not only a shift in skills but also a profound reconstruction of cognitive patterns and communication approaches [18].

Notably, role identity plays a central role in the transition process. Research indicates that athletic identity can function both as a resource and as a constraint. On the one hand, a strong athletic identity helps maintain a sense of self-worth and career continuity; on the other hand, if individuals fail to reconstruct this identity effectively, they may experience role conflict and adaptation difficulties [4]. Therefore, the transition process is essentially an ongoing process of meaning construction, in which individuals continuously reinterpret their roles and career goals through interactions with others and the environment. Although existing studies have explored athlete-to-coach transitions from multiple perspectives, they have largely focused on relatively mature institutional contexts, such as professional clubs or high-performance training environments, while paying limited attention to coaching within educational systems [12,19]. In fact, compared with elite sport systems, school sport contexts differ significantly. On the one hand, school sport not only involves athletic training but also fulfills educational functions, with core goals shifting from “performance-oriented” to “development-oriented.” On the other hand, school coaches are often required to balance multiple roles, such as educator, trainer, and manager, and this multiplicity of roles can easily lead to role conflict and identity tension. In addition, school organizational structures, evaluation systems, and interactions with students and parents create new challenges for coaches’ occupational adaptation. Existing research shows that school coaches are often underprepared in areas such as mental health support, social-emotional education, and communication skills, and face high levels of stress and turnover risk in practice [20,21].Overall, the literature on school coaches includes not only studies on athletes transitioning into coaching roles, as discussed above, but also research on school coaches generally, which has primarily examined professional socialization [22], professional development [23,24], occupational challenges [25,26], and other aspects of school coaching careers. In addition, an emerging body of research has focused on China-specific school-coach policy implementation.

To rapidly achieve international success in competitive sports, China established the Central People’s Government Sports Commission in 1952 and gradually developed the foundation of a state-led elite sport system, including professional sports teams, sports universities, and a competition system. Subsequently, the sport system gradually became separated from the education system. The long-standing separation between sport and education has generated a series of challenges, including difficulties in athletes’ career transitions after retirement, insufficient integration between elite sport development and youth development in schools, and limited opportunities for students to participate in systematic sports training. To address these challenges, China initiated reforms promoting sport–education integration in 2020 [27]. In January 2023, the General Administration of Sport of China, together with four other ministries, issued the *Opinions on Establishing Coaching Positions in Primary and Secondary Schools*, formally requiring regular schools to establish dedicated coaching positions and promoting the large-scale incorporation of retired athletes into the education system [28]. Subsequently, all provincial-level regions in China introduced corresponding implementation measures based on this policy. For example, Anhui Province, where approximately 100 athletes retire annually, established 82 dedicated positions for retired athletes in 2023, including positions as school physical education teachers and school coaches [29]. In Chinese academic discourse and in the present study, “school coaches” specifically refers to formally appointed staff members within the school system who are recruited from elite athletes after retirement. The institutional innovation of school coaching positions has not only reshaped the staffing structure of school-based physical education but has also enabled a large number of retired athletes to enter, within a relatively short period, a professional field substantially different from their previous sporting environments. Compared with existing research contexts, the career transition of school coaches in China exhibits multiple overlapping characteristics. First, as a newly established occupation, the institutional framework for school coaches remains underdeveloped, with job requirements, professional norms, and career pathways still in the process of formation, thereby increasing uncertainty in individual adaptation. Second, the transition of retired elite athletes into school coaches involves both “career transition” and “institutional migration,” requiring individuals not only to shift from athlete to coach but also to adapt across fields—from the elite sport system to the education system. The role expectations in school contexts are more complex, encompassing training, competition, education, and management. If occupational adaptation at this stage is unsuccessful, it may not only affect current job performance but also hinder long-term career development [30].

Although Chinese scholars have conducted theoretical discussions on the value of school coaching positions [31], professional development rights [32], and policy practices [33,34], empirical research based on systematic data remains limited, particularly regarding how retired athletes adapt to school coaching roles after transitioning from elite sport systems. As a major source of the school coaching workforce, retired athletes entering educational settings must reinterpret and reconstruct their occupational identities within a new institutional context. Examining their occupational adaptation is important not only for promoting the career development and well-being of school coaches, but also for advancing understanding of how emerging occupational roles are experienced and enacted in contexts of institutional transformation. Therefore, this study adopts a qualitative approach to explore the occupational adaptation experiences of retired elite athletes transitioning into school coaching positions. Specifically, focusing on school coaches with retired athlete backgrounds in China, this study aims to address two questions: how retired elite athletes experience and navigate their transition into school coaching positions; what factors shape their occupational adaptation trajectories.

### Theoretical foundation

Institutional logic theory posits that relatively stable value orientations, behavioral norms, and meaning systems exist within different institutional fields, collectively forming the basic framework through which actors understand the world and guide their practices [35]. Within the emerging occupational context of school coaches, individuals do not confront a single institutional logic; rather, they operate within a complex environment characterized by the intersection and misalignment of state logic, school logic, and professional logic. State logic is reflected in the institutional expectations generated under the policy agenda of the integration of sport and education. Under this macro-level institutional design, school coaches are assigned multiple responsibilities [28], reflecting the characteristics of role hybridity and functional integration. School logic, however, is more strongly constrained by the realities of school operations, where physical education teaching and academic advancement rates are prioritized, while training and competition may occupy a marginalized position. Professional logic, by contrast, is rooted in the experiential structures and action dispositions formed through retired athletes’ long-term engagement in competitive sport. Under the guidance of professional logic, retired athletes tend to understand and enact their roles primarily as “coaches.” The tensions among these multiple institutional logics transform occupational adaptation from a process of learning and internalizing a single norm into a process in which individuals construct meaning and make behavioral choices amid competing institutional expectations.

In research on career transition, identity is regarded as a critical mediator linking individuals and contexts [19]. Identity construction theory emphasizes that individuals do not passively accept externally imposed role expectations; rather, they continuously construct, adjust, and sustain their understanding of “who I am” through interactions with specific contexts [36]. In the transition from athlete to coach, individuals must shift from the role of the “guided” to that of the “guide.” Within school contexts, this transformation is further extended into a reconstruction of identity from a “competitive sport participant” to an “educator” [37]. This process is often accompanied by identity tension and uncertainty. On the one hand, the experiences and values embedded within athletic identity demonstrate continuity, helping individuals maintain a sense of self. On the other hand, if this identity cannot be effectively transformed, it may become a constraint on adaptation to the new role. Moreover, although institutional logics and identity tensions provide the basic context for occupational adaptation, individuals are not entirely constrained by structural conditions. Agency theory suggests that actors are capable of making choices and taking action on the basis of their experiences, motivations, and resources within existing structural conditions, thereby influencing adaptation outcomes [38]. In career transition contexts, agency does not exist independently of identity construction; rather, it is enacted on the basis of individuals’ understandings of their own roles. Different forms and degrees of agency exert important influences on identity construction and occupational adaptation outcomes.

Based on the above analysis, this study constructs an integrated analytical framework of “multiple institutional logics–identity construction–agency.” Specifically, multiple institutional logics constitute the structural context of occupational adaptation, defining the role expectations and boundaries of action that individuals confront. On this basis, individuals continuously construct and define their occupational roles through ongoing identity construction. Agency then functions as a key moderating factor, shaping individuals’ behavioral choices and modes of enactment on the basis of identity construction. Through the interaction of these three dimensions, occupational adaptation demonstrates the characteristics of a dynamic evolutionary process. Accordingly, in the empirical analysis, this study further draws upon the staged perspective of cultural transition and understands occupational adaptation as a process unfolding through different stages [39].Within each stage, the interactions among institutional logics, identity construction, and agency are examined in order to reveal the internal mechanisms and developmental pathways of occupational adaptation.

## Methods

### Study design

The present study resided within a constructivist epistemological paradigm that views knowledge of the occupational adaptation experiences of retired elite athletes transitioning into school coaching positions as socially constructed [40]. The data were collected through semi-structured interviews with school coaches working in primary and secondary schools in China. Data analysis was conducted using Reflexive Thematic Analysis (RTA), a method that emphasizes researchers’ reflexivity and critical thinking, advocating the interpretation and construction of themes through theoretical lenses to achieve analytical depth and coherence [41]. This study is reported with reference to the Consolidated Criteria for Reporting Qualitative Research (COREQ) to enhance transparency in reporting. However, methodological decisions throughout the study were guided by the philosophical assumptions and analytical principles of Reflexive Thematic Analysis rather than by checklist requirements.

### Research team

This study was conducted by two researchers. The first author is a 29-year-old female, and the second author is a 36-year-old male. The first author is a doctoral candidate at Shanghai University of Sport, and the second author is a university lecturer holding a doctoral degree. Both authors have academic backgrounds in physical education and sport training, with in-depth knowledge of school sport practices in China, the integration of sport and education reforms, and the institutional framework of school coaching positions. Both researchers have received systematic training in qualitative research and have professional experience working in educational settings. The first author previously worked as a physical education teacher in a secondary school, while the second author has experience in teaching, coaching, and administrative roles in higher education institutions. These experiences provided important contextual understanding of school-based physical education and coaching practices.

At the same time, the researchers were aware of the differences between themselves and the participants—most participants were retired elite athletes whose professional training pathways and career transition trajectories differed from those of the researchers. The researchers’ prior experiences within the physical education and sport system may have influenced their interpretation of participants’ narratives. Therefore, throughout the research process, the researchers engaged in ongoing reflexive practice, critically examining and questioning potential preconceptions and interpretive biases through team discussions and critical dialogue, in order to enhance the openness and credibility of the analysis.

### Participants

Participants were recruited using purposive sampling to ensure they were “information-rich” with respect to the research questions [42]. Specifically, participants were required to meet the following criteria: (1) having previously been registered athletes at or above the National First-Class Athlete level; (2) having transitioned into full-time, officially employed school coaches through relevant policy initiatives after retirement; and (3) having at least one year of work experience in this role. Data were collected through interviews conducted between 2025-03-04 and 2025-12-11. To capture diverse perspectives, maximum variation sampling was employed across dimensions such as age, gender, athletic level, sport type, school level (primary or secondary), and geographic region. A total of 30 school coaches participated in this study (see Table 1). To protect participants’ identities, each participant was assigned a pseudonymous code. “M” and “W” denote male and female participants, respectively, while the accompanying numbers indicate the participant sequence number. These codes were used consistently throughout the Results section to distinguish participants while maintaining anonymity.

**Table 1:** Anonymized characteristics of interview participants.

Characteristic	Category	n	%
Gender	Male	18	60.0
	Female	12	40.0
Age group	25–29 years	21	70.0
	30–34 years	9	30.0
Athletic classification	Master Athlete	20	66.7
	First-Class Athlete	10	33.3
School level of employment	Primary school	15	50.0
	Junior high school	10	33.3
	Senior high school	5	16.7
Employment region	County-level area	13	43.3
	Prefecture-level city	17	56.7
Actual tenure at interview	1.5 years	20	66.7
	2 years	10	33.3

Note. To protect participant privacy, specific participant-level identifiers, exact ages, specific sport events, and specific employment locations have been removed or aggregated. Actual tenure is reported in aggregated categories.

### Ethics statement

Ethical approval was obtained from the Ethics Committee of Shanghai University of Sport (Approval No.: 102772025RT054). Written informed consent was obtained from all participants before data collection. Participants were informed that anonymized interview data and quotations might be used in academic publications, and they provided consent for the publication of the study findings.

### Data collection

Data collection was conducted in three stages. In the first stage, the research team obtained information on the recruitment results and lists of school coaches from the official website of the Huaibei Municipal Education Bureau in Anhui Province. Field visits were then conducted in schools, and two coaches each from primary and junior secondary schools were selected. Following each interview, the researchers engaged in preliminary familiarisation with the interview material and held reflective discussions about issues emerging from participants’ accounts. These discussions were used to identify topics that required greater exploration and to refine the interview guide iteratively. The interviews from this stage were retained within the final dataset. In the second stage, purposive sampling combined with maximum variation sampling was employed to recruit additional participants. Participants were recruited through multiple sources and regions, including Huaibei City, Hefei City, Suixi County, and Linquan County in Anhui Province, Ningbo City and Shaoxing City in Zhejiang Province, and Guangzhou City in Guangdong Province. Recruitment was primarily conducted through snowball sampling, whereby initial participants recommended other eligible school coaches who met the inclusion criteria. Through this process, 20 participants were interviewed in the second stage. In the third stage, six further interviews were conducted to enhance the breadth and depth of the dataset by including additional perspectives from participants with different adaptation experiences. Throughout the recruitment process, no eligible participants refused participation or became unreachable after initial contact.

Interviews were conducted face-to-face during participants’ non-working hours. Prior to each interview, the researchers explained the purpose of the study, provided the interview guide, and obtained written informed consent from participants. During the data collection process, informal conversations also occurred between researchers and participants. These informal conversations were not included in the formal dataset or analysis. Instead, they were used solely for establishing rapport, facilitating communication, and gaining contextual understanding of participants’ occupational backgrounds and experiences. All formal interviews were audio-recorded in full. To ensure recording quality and participant comfort, interviews were conducted either in school offices or in off-campus cafés, with no unrelated individuals present. Each participant was interviewed for approximately 1.5 hours in total. The interview guide was initially developed based on literature review and exploratory interviews. Initial guiding questions included: “Can you describe your experiences from retirement to before entering your current position?” and “Can you describe your experiences after starting this job?” These were followed by key questions such as: “Have you adapted to your work after entry?”, “What challenges have you encountered?”, and “What factors have influenced your occupational adaptation?” Throughout the interview process, questions were flexibly adjusted according to participants’ responses in order to explore emerging issues in greater depth. All interviews were transcribed verbatim. To enhance credibility through member checking, interview transcripts were returned to participants for review. A total of 16 transcripts were sent back to participants, and two participants provided feedback. Their feedback mainly involved adding supplementary information regarding their employment entry experiences and requesting the removal of certain sensitive personal details. Participants were not asked to review or validate the researchers’ interpretive themes. The feedback therefore resulted in minor modifications to individual transcript content but did not alter the dataset structure, coding process, or analytical interpretations.

### Data analysis

Data were analyzed using Reflexive Thematic Analysis (RTA) as proposed by Braun and Clarke. RTA emphasizes the researcher’s ongoing reflexivity and the interpretive construction of themes based on theoretical positioning [41]. The analysis followed the six-phase process. In Phase 1 (familiarization with the data), the first author repeatedly read all interview transcripts to gain an in-depth understanding of participants’ occupational transition experiences. During this process, preliminary analytical notes were recorded regarding recurring experiences, contradictions, and meaningful events related to retirement transition, entry into school coaching positions, workplace adaptation, and identity changes. In Phase 2 (generation of initial codes), transcripts were systematically coded by identifying meaningful segments related to participants’ experiences and interpretations. The coding unit was a meaningful idea or experience rather than a predetermined linguistic unit. Coding involved both semantic and latent interpretation. At this stage, coding was primarily inductive, allowing concepts and patterns to emerge from the data rather than applying predetermined theoretical categories. In Phase 3 (development of initial themes), related codes were compared, clustered, and organized into broader patterns of shared meaning. For example, participants’ accounts of difficulties in transforming athletic expertise into school-based teaching, adapting training-oriented approaches to classroom contexts, and fulfilling new educational responsibilities were initially coded as “difficulty translating athletic expertise into teaching practice,” “mismatch between coaching experience and school teaching requirements,” and “uncertainty regarding the school coach role.” Through cross-case comparison, these codes were integrated into the initial theme of “reality shock”.

In Phase 4 (review and refinement of themes), candidate themes were repeatedly examined against coded extracts and the complete dataset to assess their internal coherence, distinctiveness, and explanatory power. During this stage, the analytical framework of “multiple institutional logics, identity construction, and agency” was introduced as a sensitizing framework to deepen interpretation. Importantly, the theoretical framework did not function as a rigid coding template applied deductively to the entire dataset. Instead, it interacted dynamically with the coding process. Emerging themes were continuously examined against both the empirical data and the theoretical framework, allowing the researchers to refine interpretations while remaining grounded in participants’ experiences. For instance, extracts initially coded as “desire to maintain coaching responsibilities,” “pressure to fulfill teaching expectations,” and “reality shock” were initially treated as separate experiences. Through iterative comparison and theoretical reflection, these patterns were interpreted as manifestations of tension between professional logic and school logic, which contributed to the development of the theme “Role Negotiation.”

In Phase 5 (defining and naming themes), the researchers clarified the central meaning and boundaries of each theme and examined how themes collectively represented the process of occupational adaptation. Through iterative comparison between empirical materials and theoretical interpretations, three overarching themes were developed: Role Pre-adaptation, Role Negotiation, and Role Orientation. These themes reflected different stages of retired athletes’ transition from elite sport environments into school coaching contexts. In Phase 6 (producing the final thematic narrative), the final thematic structure was constructed into a coherent analytical account explaining how retired athletes experienced occupational adaptation and how their adaptation pathways were shaped through interactions among institutional logics, identity construction, and agency.

The second author contributed to data familiarization, coding discussions, and theme development and refinement. Rather than seeking inter-coder agreement, differences in interpretation were treated as opportunities for deeper reflection and analytical development, consistent with the reflexive orientation of RTA. For example, during theme refinement, the researchers initially differed in interpreting participants’ accounts of difficulties in balancing coaching responsibilities and teaching expectations. The initial interpretation viewed these experiences primarily as practical challenges encountered during workplace adaptation, whereas further discussion highlighted that these accounts reflected a deeper negotiation between professional sport identity and school-based educational expectations. This discussion contributed to refining the interpretation of these experiences from isolated adjustment difficulties into the broader theme of “Role Negotiation.” Through ongoing dialogue, the researchers revisited relevant data extracts, reconsidered alternative interpretations, and refined the thematic structure. No qualitative data analysis software was used; transcripts and coding records were managed manually throughout the analytical process.

## Results

The reflexive thematic analysis generated three interconnected themes that represent the process of occupational adaptation among retired athletes transitioning into school coaching: Role Pre-adaptation, Role Negotiation and Role Orientation. Together, these themes illustrate a dynamic process through which retired athletes gradually reconstruct their occupational identities while navigating the transition from competitive sport environments to educational institutions. Table 2 provides an overview of the thematic structure, including the themes, subthemes, and their key meanings.

**Table 2:** Overview of the thematic structure.

Theme	Subtheme	Brief description
**Role Pre-adaptation: From Retirement to Acquiring Occupational Qualification**	**Occupational preparation through pre-entry training**	Prior to formal employment, retired athletes acquired preliminary knowledge of school coaching through pre-service training, internships, and transitional work experiences, which facilitated their initial understanding of school-based occupational roles.
	**Variation in pre-entry readiness**	Differences in regional training provision, employment opportunities, and individual engagement resulted in uneven levels of preparedness for entering school settings, shaping subsequent adaptation experiences.
**Role Negotiation: Occupational Practice under Reality Shock**	**Reality shock during occupational transition**	Participants experienced discrepancies between expectations formed during athletic careers and the realities of school work, particularly regarding teaching responsibilities, classroom management, administrative tasks, and educational objectives.
	**Negotiating occupational identity**	Participants continuously reinterpreted the meaning of being a school coach while balancing expectations associated with competitive sport and school education, leading to ongoing identity adjustment.
	**Reconstructing interpersonal relationships**	Occupational adaptation required participants to develop new patterns of interaction with students, colleagues, school leaders, and parents, replacing the hierarchical relationships typical of elite sport with more collaborative educational relationships.
**Role Orientation: Differentiation of Occupational Adaptation Outcomes**	**Institutional embedding and career development**	Institutional arrangements, including professional evaluation systems, promotion opportunities, coaching responsibilities, and school support, shaped participants’ long-term occupational trajectories and adaptation outcomes.
	**Role integration**	Participants successfully integrated their identities as both coaches and educators, combining competitive sport expertise with educational responsibilities to develop a stable occupational identity.
	**Role assimilation**	Participants gradually abandoned the coaching-oriented dimension of their work and increasingly identified themselves as ordinary physical education teachers, with coaching identity becoming weakened or dissolved.
	**Role alienation**	Participants failed to establish either a stable coach identity or a strong sense of belonging within the school system, resulting in occupational uncertainty, low identification, and, in some cases, intentions to leave the profession.

### Role pre-adaptation: From retirement to acquiring occupational qualification

Before formally entering school coaching positions, retired elite athletes typically undergo a transitional stage of “role pre-adaptation.” This stage is not merely a period of waiting or preparation; rather, it is a process in which individuals, prior to fully entering a new occupational context, develop preliminary understandings and construct meanings regarding their future occupational roles through prior experiences and limited forms of practice. Following retirement, interviewees commonly encountered one or more of three transitional situations before entering school coaching positions. The first involved temporarily remaining unemployed at home, either to recover physically or to search for employment opportunities. The second involved taking temporary jobs either related or unrelated to their original sport specialization. The third involved participating in institutionalized preparatory activities, such as pre-service training programs or job-shadowing internships for school coaches. In comparison, pre-service training and job-shadowing internships constituted the key experiences for most retired elite athletes prior to entering school coaching positions. Because some regions explicitly required applicants to obtain relevant training certificates before employment [43], the vast majority of interviewees participated in different forms of pre-service training. Twenty-eight participants (93.3%) had completed pre-service training, and 14 participants (46.7%) had participated in job-shadowing internships. All participants who had completed job-shadowing internships (n = 14) had also completed pre-service training. In contrast, two participants (6.7%) had completed neither pre-service training nor job-shadowing internships. These programs not only provided basic teaching knowledge and institutional norms, but also enabled retired elite athletes to encounter school contexts in a limited capacity and develop preliminary understandings of their future work:

*“Before entering the job, there were two rounds of training. One was a job-shadowing internship, where we followed PE teachers in schools, learned how they taught classes, took notes, and trained for one week. Then in July, there was another month of centralized training. These trainings were quite useful for me. At the very least, I learned basic classroom routines. If I had retired and gone directly into a school, I probably wouldn’t have known anything… After the training, I became quite clear about classroom routines in PE and the basic content of lesson plans” (M10).*

In addition to institutionalized training, some interviewees worked in youth sports clubs and related settings after retirement. These experiences likewise provided important references for understanding “how to interact with students” and “how to conduct daily management”:

*“Before starting the job, I worked in a sports club for two years. Those experiences were actually helpful for my current work at school, especially in managing and communicating with children. The kids in the club were about the same age as junior high school students. I spent two years with them, living at the club almost every day and rarely going home. This experience helped me better understand children’s thoughts” (M3).*

Whether through temporary employment or institutionalized training, these experiences were not merely preparations at the level of skills, but also processes through which individuals continuously revised their understandings of the school coach role across different contexts. Notably, however, the effectiveness of this pre-adaptation stage varied considerably. Significant regional differences existed in terms of training organization, curriculum content, and training duration. For example, training programs in some regions lasted several months (e.g., Hebei), whereas in others they lasted only about one week (e.g., Guangxi and Fujian) [44,45]. In some regions, the training content also suffered from excessive theoretical emphasis and insufficient integration between courses, thereby limiting learning effectiveness:

*“The pre-service training lasted nearly a month. Every morning classes started at 8:30 and continued until 11:30, with only enough time for us to go to the bathroom and drink some water. Then classes resumed at 2:30 in the afternoon and lasted until after 5. In the evening, we gathered again after 7 to reflect and write notes until after 9. During that period, I felt mentally overwhelmed. The schedule was too tight, and everyone was really exhausted” (M30).*

*“Although the course content was very rich, most of it was theoretical. Some teachers went too deep and too theoretical. Since we had basically never experienced systematic academic learning, it was difficult for us to fully understand. For example, when they explained the new curriculum standards, it was too broad. I needed more detailed and practical things that I could directly apply” (W7).*

Even under the same institutional arrangements, individuals’ levels of initiative and reflection during participation significantly influenced the effectiveness of role pre-adaptation. Some interviewees regarded the training as a “mandatory procedural task,” believing that their competitive sport experience alone was sufficient for school sport work, and therefore participated rather passively:

*“I felt that my competitive sport experience was already rich enough, so I didn’t really want to attend the training. But if you didn’t go, you couldn’t officially take the job. At that time, I was still working as a substitute teacher elsewhere, but after considering it, I eventually decided to attend” (W24).*

*“I don’t think many people were genuinely interested in learning. For me, it didn’t have much effect, and I don’t remember much of the training content now” (M19).*

Overall, although the pre-adaptation stage formally provided retired elite athletes with a “qualification pathway” for rapidly entering the school coaching occupation, in substance it more closely resembled an incomplete process of role rehearsal. While individuals acquired occupational qualifications in an institutional sense, their understanding of the occupational role remained preliminary and unstable. This state of “being qualified but not yet truly understanding the role” laid the groundwork for the adaptation shocks they would later encounter upon entering real work contexts.

### Role negotiation: Occupational practice under reality shock

After formally entering school coaching positions, retired athletes’ process of occupational adaptation moves into a highly dynamic and tension-filled stage of role negotiation, particularly during the first semester when they encounter profound “reality shock.” Many interviewees experienced adaptation difficulties across multiple dimensions. In terms of occupational skills, although retired athletes possessed high levels of competitive expertise and had undergone pre-service training, they still faced significant challenges in physical education teaching:

*“To be honest, I knew I wasn’t very good at teaching skills”(M5).*

*“PE classes were quite different from what I had imagined. I originally thought students would follow instructions and behave obediently, but in reality they were much harder to manage than I expected… I still remember the first time I taught a class—my whole body was soaked with sweat. The rhythm of teaching was completely different from that of sports training, and I was extremely nervous” (W11).*

Many school coaches also encountered substantial difficulties when dealing with work-related textual tasks, such as writing lesson plans, completing classroom observation records, and preparing semester summaries:

*“I had never written lesson plans before, so I really didn’t know how to do it. Every semester we also have to submit classroom observation records and semester summary materials, which is really troublesome” (M1).*

The normative requirements embedded within the school context further intensified the occupational adaptation difficulties faced by school coaches. A particularly typical example was the system of open classes, which emphasizes standardized teaching procedures and instructional methods. This differed substantially from the task-oriented training approaches to which retired athletes were accustomed. Mastering these norms within a short period posed considerable challenges for most newly appointed coaches:

*“I spent more than a week preparing for the open class, and it felt quite difficult. In ball sports, the principle is that the ball should never leave the players’ hands, meaning ideally every student should have a ball. But in regular classes that’s unrealistic. However, open classes require it. When everyone has a ball, the scene becomes chaotic, and I couldn’t control it well” (M4).*

Although some schools provided support through measures such as mentorship programs, the effectiveness varied considerably. In most cases, institutional support failed to function fully, and individuals had to rely on their own initiative to complete skill transformation:

*“My mentor teaches around 15 classes a week and doesn’t really have time to observe your lessons. Although mutual classroom observation is required by policy, in reality you mostly have to rely on yourself” (M22).*

Within this context, whether individuals actively sought support directly affected their adaptation process:

*“Whenever I encountered problems, I liked to ask my mentor. He’s a really kind person. Although he has administrative duties, every time I asked him questions, he patiently helped me. So I adapted to PE teaching very quickly—basically within about two months after entering the job, I was already teaching quite well” (W7).*

At the same time, some individuals, despite recognizing their own lack of competence, did not take proactive action:

*“For PE classes or other sport-related skills, I didn’t deliberately try to learn more. After work, I did things I enjoyed, such as watching variety shows or anime” (W24).*

In addition to challenges arising from specific work tasks, school coaches also needed to bridge the gap between the occupational cognition they had formed before employment and the realities of the school context. Having undergone many years of professional athletic careers, school coaches possessed strong emotional attachments to their sports and expected to continue utilizing their expertise, viewing “training and organizing competitions” as the core content of their future work:

*“I practiced handball for more than ten years, so it’s impossible not to have feelings for it. After entering the school, I wanted to lead team training. Becoming an excellent handball coach has always been my ideal” (M20).*

However, after entering school positions, all school coaches discovered that their work primarily revolved around physical education teaching. In some schools, training conditions were even absent altogether, and half of the interviewees reported having no coaching-related responsibilities at all:

*“After entering the job, there were no coaching-related tasks at all. Every day was just about teaching PE classes” (W27).*

Due to the gap between occupational expectations and post-entry realities, some school coaches gradually accepted the identity of a “pure PE teacher” and began to clarify their responsibilities and work goals within this role:

*“I feel that I’m now simply a PE teacher… When I first entered the school, I didn’t know the requirements of the high school entrance PE examination. I thought students just practiced by themselves after class. But later I realized there were strict requirements, and then I understood that my responsibility was to improve their PE examination scores” (M5).*

In contrast, other school coaches underwent a cognitive shift from a “single coaching responsibility” to a “multi-dimensional educational responsibility.” They regarded physical education teaching and sports training alike as part of their responsibilities and directions for future effort, and acted accordingly:

*“I think teaching PE well and achieving good training results are both very important. But right now, the main problems in specialized training are the lack of funding and facilities. This semester, I plan to first carry out some basic training temporarily on the athletics field. Later, when we move on to technical training, I’ll have to find ways to secure more professional facilities” (M1).*

In addition, changes in interpersonal interaction patterns also constituted an important aspect of role negotiation. Long-term experiences in sports teams had accustomed school coaches to interacting with students through authoritative and command-based approaches. However, within school contexts, such interaction patterns often proved ineffective, especially when dealing with younger students:

*“Teaching PE to first-, second-, and third-grade students is very difficult. They are too young, and their psychological or cognitive development may still be incomplete. Communication with them is really difficult. Next semester, I only want to teach higher grades” (W29).*

Through ongoing practice, some interviewees gradually began to actively adjust their interaction styles with students:

*“If you speak to students in a strict tone, they will listen at that moment, but it doesn’t last long. Later, I borrowed a method from my part-time work before employment, using star stickers. Whoever performed well in class would receive a star sticker recorded in their notebook, and after collecting a certain number, they could exchange them for rewards. It worked very well” (M5).*

Occupational adaptation during the initial stage of employment represented a process of role negotiation triggered by reality shock. During this process, retired athletes’ previous experiences, professional identities, and understandings of coaching were continuously negotiated within the institutional context of schools. Through ongoing practice, reflection, and interaction with colleagues and students, some individuals, such as M5, gradually redefined the meaning of the “school coach” role. After approximately one academic semester (about four months) of employment, participants had become increasingly familiar with their job requirements; however, their understandings of the role remained open to further adjustment and reconstruction. This ongoing process of role negotiation extended into the subsequent stage of role orientation.

### Role orientation: Differentiation of occupational adaptation outcomes

As the reality shock experienced during the initial stage of employment gradually subsided, retired athletes entered the stage of “role orientation” through ongoing occupational practice and interaction. During this stage, school coaches no longer faced severe difficulties in occupational skill adaptation, but instead encountered challenges related to interpersonal relationship reconstruction, institutional embedding, and career development pressures. In terms of interpersonal adaptation, the focus gradually shifted from interactions with students to relationships with personnel within the school organization. Compared with classroom teaching interactions, relationships with colleagues and school administrators were characterized by greater uncertainty and complexity:

*“Social interactions with colleagues and school leaders are more complicated than the actual work tasks themselves” (M16).*

Some interviewees gradually developed tendencies toward avoidance in their interactions with colleagues:

*“Some colleagues are much older and not easy to communicate with… During that period, my emotional state was really poor” (W17).*

At the same time, some interviewees gradually established new organizational relationship networks through proactive communication and participation in school activities:

*“When I was an athlete, I mostly interacted with teammates and coaches, and rarely socialized with other people. So when I first came here, I wasn’t very good at dealing with others. Later, I realized this wouldn’t work, so I started actively chatting more with colleagues around my age, asking them questions, and participating in school activities whenever possible. Eventually, once everyone became familiar with each other, things improved” (M26).*

With regard to institutional embedding, teacher certification requirements and professional title evaluation systems became important factors shaping occupational adaptation. At present, retired athletes entering school coaching positions are not legally required to possess teaching certificates before employment. However, because physical education teaching constitutes one of their core responsibilities—and Chinese law stipulates that individuals engaged in teaching must hold teaching qualifications—many retired athletes obtain certification only after entering the profession. If school coaches do not possess a teaching certificate, or if their certificate level is lower than that required by the school level in which they work (for example, W7 and M10 only held primary school teaching certificates while working in high schools), schools may encounter compliance difficulties when arranging teaching assignments:

*“The school required me to obtain a teaching certificate within two years; otherwise, they would have difficulties assigning me classes. I had no choice but to spend a lot of time preparing for the exam after classes and after work, which felt very challenging” (W29).*

More seriously, in many provinces in China, professional title evaluation systems specifically designed for school coaches have not yet been fully established. As a result, school coaches can only apply for professional titles under the evaluation standards for physical education teachers:

*“Professional title evaluations can only follow the standards for PE teachers because the evaluation scheme specifically for school coaches has not yet been finalized” (W15).*

Although a few provinces have already introduced professional title evaluation schemes for school coaches, these policies largely mirror those designed for professional sports coaches. In other words, sending athletes to higher-level training organizations and achieving competitive results constitute the primary criteria for career advancement. Consequently, many school coaches who do not have opportunities to lead sports teams experience severe career development crises:

*“It’s extremely difficult to obtain professional title promotion. You have to find ways to send students into provincial teams, and after entering the provincial team they must become officially registered athletes and achieve national-level results before it counts as my achievement. Only then can I apply for promotion. The whole process takes many years. Besides, I don’t even have a single athlete under my supervision right now, so career advancement feels indefinitely out of reach” (M16).*

National policy stipulates that school coaches and physical education teachers may transfer into each other’s positions once relevant conditions are met. This means that school coaches may also pursue professional title promotion through the evaluation system for PE teachers. Consequently, many interviewees expressed intentions to transfer into PE teaching positions in order to achieve career advancement through the teacher evaluation system:

*“Using the teacher evaluation system definitely gives you a better chance. Teacher title evaluations happen once every year, and there are quotas for secondary- and first-level teachers at the county level, so it feels more controllable. In the future, I still hope to transfer into a teaching position” (M30).*

Many school coaches without coaching responsibilities remain dependent on the existing institutional system for physical education teachers in terms of career development. They must not only adapt to teaching work, but also continuously adjust their career plans in accordance with the operational logic of school institutions. Within this process, occupational adaptation outcomes gradually diverged across individuals in both occupational practice and occupational cognition, eventually stabilizing into three relatively distinct pathways: role integration, role assimilation, and role alienation. Table 3 summarizes the defining indicators, representative cases, and discrepant or borderline cases associated with each pathway.

**Table 3:** Characteristics of occupational adaptation pathways.

Pathway	Defining indicators	Representative cases (borderline cases)
**Role integration**	(1) Maintaining both coaching and educational identities; (2) integrating competitive sport expertise with school-based educational responsibilities; (3)developing a stable occupational identity as a school coach.	W15, W18, M1, M26 (No borderline cases)
**Role assimilation**	(1) Limited or absent coaching responsibilities; (2) work content and developmental pathways gradually converge with those of ordinary PE teachers; (3) increasing self-identification as a PE teacher rather than a school coach.	W2, W27, M5, M30 (W29 and M3 demonstrated characteristics of role assimilation to some extent; however, they still aspired to return to coaching responsibilities or pursue career development through coaching achievements.)
**Role alienation**	(1) Difficulty integrating into the school teacher system while also failing to genuinely acquire a coach identity; (2) weak occupational identification; (3) uncertainty regarding future career development and possible intention to leave the occupation.	M6, W24, W17 (No borderline cases)

Role integration refers to individuals’ ability to reconstruct the meaning of the “coach” role within the educational context of schools, effectively integrating competitive sport experience with educational responsibilities. Individuals following this pathway are capable not only of undertaking physical education teaching tasks, but also of actively seeking opportunities for sports training, thereby developing relatively stable occupational identities through practice, ultimately achieving relatively high levels of occupational adaptation:

*“Right now, I both coach the school team and teach PE classes. I feel these two identities can actually be integrated together” (W15).*

*“Gradually, I realized that training in schools is different from training in professional teams. You can’t only focus on competitive results; you also have to consider students’ interests and academic situations. Now I actively communicate with homeroom teachers about students’ conditions and adjust my training methods accordingly. I feel that I’ve already adapted to the role of school coach” (W18).*

Role assimilation, by contrast, refers to individuals gradually abandoning their original orientation toward competitive sport and fully embedding themselves within the school teacher system. During this process, the identity of “coach” becomes weakened or even dissolved, and individuals increasingly define themselves as “physical education teachers.” Their work content and developmental pathways gradually converge with those of ordinary PE teachers:

*“I don’t have any training responsibilities. Every day I just teach PE classes, supervise exercise breaks, and complete tasks assigned by the school. After a long time, I feel that I’m no different from an ordinary PE teacher” (W2).*

Role alienation refers to situations in which individuals are unable to integrate into the school teacher system while also failing to genuinely acquire a coach identity, thereby developing a strong sense of occupational alienation. These individuals typically experience high levels of pressure and low levels of occupational identification, with some even showing tendencies toward resignation. Their occupational adaptation processes thus exhibit clear characteristics of obstruction:

*“Sometimes I feel that I don’t really have much presence in the school, and the school itself doesn’t place much importance on sport. Back when I was in the sports team, at least I knew clearly what my goals were. But now I often feel lost, and I’ve even thought about whether I should continue doing this in the future” (M6).*

## Discussion

### Occupational adaptation as a staged process of identity construction

Career transition is not merely a process of competence transfer or job change, but also one of identity reconstruction, self-meaning adjustment, and role redefinition [4]. Scholars such as Chroni and Leeder have emphasized that the core challenge faced by retired elite athletes transitioning into coaching roles often lies not in insufficient technical expertise, but in how individuals detach themselves from their “athlete” identities and gradually establish new coaching identities [18,46]. The present study responds to these arguments and further reveals that, within the school context, the occupational adaptation of retired elite athletes is not a one-time role transition, but rather a staged process of identity construction.

Specifically, the transition of retired elite athletes into school coaching positions unfolds through three stages: role pre-adaptation, role negotiation, and role orientation. The stage of role pre-adaptation is not simply a process of obtaining institutional qualifications; rather, it is an important stage in which individuals begin to develop imaginaries of their future occupational roles. Whether through pre-service training, job-shadowing internships, or work experiences in youth sports clubs, these experiences essentially constitute contexts of “limited participation.” Although many individuals believed that their competitive sport experience would enable them to perform the job successfully, their understandings of the school coach occupation remained largely grounded in their prior experiences in competitive sport. This finding is broadly consistent with Kavanagh’s research on athlete-to-coach transitions, which suggests that many retired elite athletes initially assume that competitive sport experience can be directly transferred into coaching practice [17]. However, such role understandings, rooted in prior experience, are often challenged once individuals formally enter school settings.

After entering the profession, school coaches generally encounter “reality shock,” thereby moving into the stage of role negotiation. Existing studies have categorized athlete-to-coach transitions into “active” and “passive” pathways [13]. Retired elite athletes in China more closely resemble the “passive” type, as most had not planned to enter schools before retirement. Short-term pre-service training is insufficient to ensure occupational competence, and their entry into schools resembles a policy-driven “fast track” [14,15]. Previous research has shown that athletes transitioning into coaching roles often face changes in interpersonal relationships and role responsibilities [1]. Within school contexts, however, these changes are more profoundly reflected in the transformation of occupational identity logics. Long-term experiences in competitive sport training have accustomed retired elite athletes to understanding the “coach” role through performance-oriented, task-oriented, and authority-based approaches. In contrast, school sport places greater emphasis on educational values, normative requirements, and student development. As a result, individuals’ prior occupational experiences cannot be directly translated into effective practices within school settings, and their pre-existing role cognitions are continuously challenged by the realities of the institutional environment.

As individuals gradually become familiar with the operational logic of schools, their occupational adaptation enters the relatively stable stage of role orientation, during which different forms of occupational practice and identity gradually emerge. As identity construction theory suggests, identity is not fixed, but is continuously reconstructed and maintained through interactions within specific contexts [47]. Some individuals are able to integrate the coaching role with educational responsibilities, thereby developing relatively stable occupational identities. Others gradually abandon their original orientation toward competitive sport and come to define themselves entirely through the identity of “physical education teacher.” Still others remain unable to establish stable identification within the institutional environment of schools, ultimately developing feelings of occupational alienation. The present study extends existing understandings of “adaptation” within career transition research. Previous studies have often conceptualized occupational adaptation as a process through which individuals meet job requirements and accomplish role transition [48,49]. However, within the emerging occupational context of school coaches, occupational adaptation should instead be understood as a staged process of identity construction.

### Occupational adaptation under the misalignment of multiple institutional logics

The adaptation difficulties faced by retired elite athletes transitioning into school coaching positions stem not merely from insufficient individual competence or lack of experience, but more fundamentally from the misalignment among multiple institutional logics. Institutional logics refer to the historical patterns of socially constructed material practices, assumptions, values, beliefs, and rules through which individuals produce and reproduce material reality, organize time and space, and provide meaning to social reality [35]. Institutional logics simultaneously encompass structural, normative, and symbolic dimensions of order, while also possessing a dual character: they both constrain behavior and provide opportunities for agency and change [50]. Based on the findings of this study, the occupational adaptation process of school coaches is shaped by the misalignment among state logic, school logic, and professional logic.

State logic is reflected in the institutional expectations imposed by the government regarding school coaches. Relevant policies not only require school coaches to undertake sports training responsibilities, but also emphasize their participation in physical education teaching, competition organization, reserve talent cultivation, and the development of campus sports culture [28]. In practice, school coaches are positioned as hybrid occupational actors with multiple attributes. Compared with traditional professional team coaches, their occupational goals extend beyond competitive performance to encompass students’ holistic development and school sport governance [51]. However, once this macro-level institutional design enters the organizational level of schools, it fails to produce stable and coherent mechanisms of practice. School logic, by contrast, is more strongly constrained by practical operational conditions. Within the current basic education system, schools continue to operate primarily around teaching quality and examination-oriented advancement. As a result, physical education classes, student management, and the organization of school sports activities constitute the core components of school sport work [52]. Consequently, although many schools have institutionally introduced the position of “school coach,” in actual practice they tend to absorb retired elite athletes into the existing system of physical education teachers. This means that, under school logic, the identity of the school coach is continuously “teacherized,” with its occupational distinctiveness weakened or even assimilated into the pre-existing teacher institutional system. This finding is broadly consistent with previous research on organizational isomorphism in schools, which suggests that schools, as typical educational organizations, tend to organizationally absorb newcomers through existing institutional norms and operational routines, gradually aligning them with established role orders and working practices [53,54].

At the same time, the professional logic carried by retired elite athletes through their backgrounds in competitive sport continues to shape their occupational adaptation process. Existing research has shown that athletic identity often occupies a central position in individuals’ self-concepts, while the values and behavioral patterns formed through sport possess strong continuity [6,55]. Long-term experiences in competitive sport training not only shape individuals’ occupational ideals as coaches, but also cultivate action dispositions centered on performance orientation and competitive achievement. This professional logic is reflected not only in individuals’ prior experiences and behavioral patterns, but is also embedded within the professional evaluation systems for school coaches. According to relevant reforms of professional title evaluation systems, the rank structure and promotion standards for school coaches are largely aligned with those of professional sports coaches, with core evaluation criteria primarily focused on competitive achievements such as competition results and the number of athletes transferred to higher-level training organizations [56]. This means that, although school coaches are situated within the school education system, their career development remains deeply influenced by the competitive sport system. As a result, school coaches are effectively trapped within a situation characterized by the coexistence of “educationalized work goals” and “sports-oriented evaluation goals.”

The three institutional logics differ in terms of goal orientation, behavioral norms, and role expectations, yet no stable coordination mechanism has emerged among them in actual practice [57], thereby creating a “misaligned institutional context.” The position of school coach in China did not emerge organically through professional evolution. Rather, it represents a new institutional arrangement embedded by the education system within the existing teacher staffing framework under the policy background of the integration of sport and education [37]. Consequently, after entering schools, retired elite athletes are not merely adapting to institutions; through their concrete actions, they are also participating in the practical construction of institutional meaning. Occupational adaptation among school coaches is therefore no longer simply a matter of integrating into existing institutions or learning established occupational norms, but instead involves a process in which individuals reinterpret their roles and make behavioral choices amid conflicting institutional expectations.

### Agency intervention and the differentiation of occupational adaptation pathways

Although school coaches generally face similar occupational environments and institutional constraints, different individuals ultimately develop three differentiated outcomes of occupational adaptation: role integration, role assimilation, and role alienation. Existing research has suggested that career transition is not a process in which individuals passively accept environmental discipline, but rather one involving active identity negotiation and meaning construction [58]. In the present study, the occupational adaptation of school coaches cannot be understood merely as a passive outcome produced by external factors. How individuals respond to institutional tensions and take action also profoundly shapes the direction of occupational adaptation. When confronted with problems such as insufficient training resources, ambiguous role boundaries, and restricted career development opportunities, some school coaches did not remain confined within their prior competitive sport experiences. Instead, they actively adjusted their understandings of the school coach role. They gradually learned to navigate school rules, modify their interaction patterns with students and colleagues, and attempt to establish new logics of action between competitive training and educational requirements. This process of adaptation did not involve a complete abandonment of their prior identities, but rather constituted a continuous process of identity integration. As previous studies have emphasized, successful career transition does not necessarily imply the disappearance of prior identities; rather, it involves individuals reassigning new meanings to existing experiences within new contexts [59].

At the same time, some school coaches gradually abandoned their expectations associated with the “coach” identity and instead actively aligned themselves with the school teacher system. This pathway should not simply be interpreted as maladaptation, but rather as a form of realistic institutional response. Given the relative lack of training resources, career advancement opportunities, and organizational support within schools, some individuals gradually realized that relying solely on the identity of “school coach” could not provide stable career expectations. Consequently, they began proactively obtaining teaching certificates, accumulating teaching achievements, and gradually replanning their career trajectories according to the logic of physical education teachers. This indicates that individuals were not merely passive recipients of institutional arrangements; rather, after assessing organizational environments and occupational opportunities, they actively adjusted their strategies. Previous research has suggested that when original occupational identities fail to receive stable institutional support, individuals often maintain career continuity by reattaching themselves to new organizational identities [60]. The pathway of “role assimilation” identified in this study essentially reflects individuals’ active reconstruction of identity under conditions of institutional uncertainty.

In contrast, “role alienation” more strongly reflects individuals’ failure to effectively complete identity adjustment. Some school coaches continued to maintain strong identification with their identities as competitive sport practitioners, yet lacked the ability to transform these identities into practical resources within school contexts. They found it difficult both to genuinely identify with the logic of school education and to sustain their previous expectations associated with professional coaching careers. As a result, they gradually fell into states characterized by blurred occupational meaning and weakened organizational belonging. Existing research suggests that one of the core risks in career transition lies in individuals’ inability to establish continuous identity meaning within new contexts. When prior identities gradually lose effectiveness while new identities fail to stabilize, occupational adaptation may easily transform into a persistent condition of suspended identity [61]. Some school coaches in this study were effectively caught in such an intermediate state of being “neither truly coaches nor fully teachers.”

The outcomes of school coaches’ occupational adaptation are embedded within diverse institutional contexts, including variations in school environments, regional development conditions, the availability of sports teams and facilities, and opportunities for professional development. However, differences in their occupational adaptation pathways are also closely associated with individuals’ agentic engagement in interpreting, negotiating, and reconstructing their occupational roles. Within the same occupational field, individuals do not inevitably arrive at identical adaptation outcomes; rather, based on their occupational expectations, experiential structures, and behavioral choices, they respond differently to institutional environments. Occupational adaptation should therefore not be understood as a process in which institutions unilaterally shape individuals, but instead as a dynamic process in which individuals continuously engage in identity negotiation, meaning interpretation, and behavioral adjustment under institutional constraints.

## Limitations

This study has several limitations. First, the interpretive influence of the researchers’ own backgrounds in the Chinese physical education and sport system, the translation of data into English, and the strong policy specificity of the Chinese context should be acknowledged. These factors may have influenced the interpretation of findings and may condition the transferability of the proposed model to other systems. Second, this study relied primarily on coaches’ self-reported interview accounts. Therefore, interpretations concerning school organizational practices, institutional arrangements, and policy implementation should be understood as reflecting participants’ lived experiences rather than comprehensive evaluations of organizational or policy processes. Third, possible social-desirability effects from school-based interviews should be acknowledged. Participants might have moderated their expressions of institutional challenges, organizational constraints, or dissatisfaction due to their ongoing employment relationships and professional identities. Finally, the cross-sectional nature of the data limited the ability to capture the dynamic evolution of occupational adaptation over time. Although participants retrospectively described their transition experiences in terms of different stages, these narratives may have imposed a staged structure on experiences that were, in practice, more dynamic and nonlinear.

## Conclusion

This study, based on the integrated analytical framework of “multiple institutional logics–identity construction–agency,” explored the occupational adaptation process among retired elite athletes transitioning into school coaching positions. The findings suggest that the occupational adaptation of school coaches is not merely a process of job integration or competence transformation, but rather a continuous process of occupational identity construction shaped by multiple institutional logics. Specifically, the transition of retired elite athletes into school coaching positions unfolds through three stages: role pre-adaptation, role negotiation, and role orientation. During the stage of role pre-adaptation, individuals develop preliminary imaginaries of the school coach role through pre-service training and job-shadowing internships, although their occupational cognition continues, to some extent, to reflect the logic of competitive sport. After entering schools, individuals continuously encounter reality shock within educational contexts, generating tensions between their prior competitive sport experiences and the educational environment, thereby moving into a stage of role negotiation centered on the meaning of their occupation. As occupational practice continues, individuals gradually diverge in terms of occupational cognition, action strategies, and identity understandings, ultimately forming three occupational adaptation outcomes: role integration, role assimilation, and role alienation. Differences in these occupational adaptation outcomes are shaped not only by individual agency, but also by the combined influence of state logic, school logic, and professional logic.

The position of school coach in China represents a new institutional arrangement embedded within the education system under the policy background of the integration of sport and education. Its role boundaries and operational rules remain in an ongoing process of construction. Consequently, retired elite athletes entering these positions do not encounter a stable and clearly defined institutional environment, but rather an institutional field characterized by uncertainty and negotiation. Their occupational adaptation process therefore reflects not only integration into existing institutions, but also responses to institutional ambiguity and forms of practical adjustment. The findings of this study provide new insights for the literature on elite athletes’ career transition and occupational adaptation, while also offering a useful perspective for understanding the emergence and practice of new occupational roles within contexts of institutional reform. Future efforts should further improve the working mechanisms and evaluation systems for school coaches. Policymakers should establish clearer role definitions, career development pathways, and evaluation standards for school coaches to reduce role ambiguity and strengthen professional identity. Schools should provide sustained organizational support, including mentoring, collaborative opportunities with physical education teachers, and access to adequate coaching resources and facilities, to facilitate role negotiation and occupational integration. In addition, training providers should move beyond short-term qualification-oriented programmes and offer systematic pre-service and in-service training that addresses pedagogical knowledge, classroom management, communication skills, and the integration of coaching expertise with educational practice. Such measures would better support the long-term professional development and well-being of school coaches while promoting the institutionalization and professionalization of this emerging occupation.

## Supporting information

S1 File Semi-structured interview guideline. The file contains the semi-structured interview guideline used for data collection in this study.
(DOCX)

S2 File COREQ checklist. This file contains the Consolidated Criteria for Reporting Qualitative Research (COREQ) checklist used in this study.
(PDF)
