# Supplementary material for: Occupational adaptation of retired elite athletes transitioning into school coaches in China
Source: PLoS One. 2026 Oct 5;21(10):e0358744. doi: 10.1371/journal.pone.0358744 (PMC13637880; doi:10.1371/journal.pone.0358744)
Supplement: Supplementary file 2 [file pone.0358744.s001.docx]

**Semi-structured interview guideline**

**Table A1.** Interview Framework and Sample Questions

| Guiding Questions | Follow-up Questions |
| --- | --- |
| Please tell me about your experiences from retirement to the period before taking up your position | Before formally taking up this position, what experiences did you have after retiring from elite sport?  Prior to your appointment, did you have any work experience related to this position? |
| Please describe your experiences after assuming your role | Have you adapted to your work after taking up the position?  What challenges have you encountered since entering the school?  In your view, what are the key skills required for your current position?  Has your understanding of the profession of “school sports coach” changed before and after entering this position? If so, what factors contributed to this change?  In this role, what do you consider to be the most important responsibilities?  After starting this job, were there any experiences or events that left a particularly strong impression on you?  Do you experience stress in your work? If so, how do you usually cope with or manage this stress? |
| What factors do you feel have affected your professional adaptation? | What factors do you think have facilitated your professional adaptation to this role?  What factors do you think have hindered your professional adaptation? How do you perceive or interpret their impact?  Have you encountered any difficulties in your work? If so, what were the main reasons, and how did you deal with these difficulties?  Before entering the school system, did you have any expectations regarding the profession of school sports coach? If so, what were they, and to what extent do these expectations align with your current experience? |
